# Kasabach-Merritt Syndrome in an Adult

**DOI:** 10.4274/tjh.2017.0429

**Published:** 2018-08-05

**Authors:** Kolar Vishwanath Vinod, Joseph Johny, Mehalingam Vadivelan, Abdoul Hamide

**Affiliations:** 1 Jawaharlal Institute of Postgraduate Medical Education and Research (JIPMER), Department of General Medicine, Pondicherry, India

**Keywords:** Hemangioma, Thrombocytopenia, Coagulopathy, Consumption

## To the Editor,

Kasabach-Merritt syndrome (KMS) is characterized by capillary hemangiomas and consumptive thrombocytopenia and coagulopathy, and may also be associated with microangiopathic hemolysis [1]. KMS is most commonly reported in infants and young children. Here we report a rare case of adult KMS in a 47-year-old woman, giving rise to severe thrombocytopenia and bleeding.

**Figure 1:**
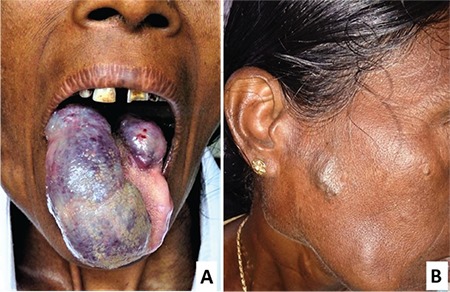
Two large purple hemangiomas of sizes 3x8 cm and 2x3 cm on the dorsum of the protruded tongue (A) and a smaller cutaneous hemangioma on the right side of the face (B).

A 47-year-old woman presented with purpuric hemorrhages over her upper and lower limbs and gum bleeds for 8 days. She was found to have two large purple hemangiomas on the tongue (Figure 1A) and a few smaller cutaneous hemangiomas on the face (Figure 1B). These lesions had been present since her childhood and the tongue hemangiomas had enlarged over the past several years. Laboratory evaluation revealed hemoglobin of 89 g/L, leukocyte count of 9.48x10^9^/L (leukocyte differential: neutrophils 72%, lymphocytes 25%, eosinophils 2%, basophils 1%), platelet count of 6x10^9^/L, prothrombin time of 13.4 s (control: 13 s), activated partial thromboplastin time of 32 s (control: 29 s), serum fibrinogen of 1.36 g/L, elevated fibrin degradation products, positive D-dimer, and normal liver and renal functions. A peripheral blood smear showed normocytic normochromic erythrocytes and markedly reduced platelets. Bone marrow evaluation revealed normal erythropoiesis, myelopoiesis, and megakaryocytic hyperplasia. Contrast-enhanced computed tomography of the chest and abdomen excluded deep-seated visceral hemangiomas. She received platelet transfusions and oral tranexamic acid for control of gum bleeds, and she was also started on oral prednisolone at 1 mg/kg/day. One month after starting the steroid treatment, the bleeding had stopped and the platelet count had improved to 152x10^9^/L. However, the hemangiomas had remained the same. Considering the risk of traumatic bleeding, she was advised to have surgical excision of the tongue hemangiomas. However, she was unwilling to undergo surgery.

KMS is most commonly reported in infants and only a small percentage (~0.3%) of infants with hemangiomas develop KMS [1]. If not recognized and treated in time, KMS may be potentially fatal by causing disseminated intravascular coagulation and severe bleeding, and large hemangiomas can cause high-output cardiac failure and vital organ compression. Though rare, KMS has been reported among adults as well [2,3,4,5]. The pathogenesis involves activation and consumption of platelets and clotting factors inside hemangiomas, giving rise to consumption coagulopathy and bleeding. However, no correlation has been reported between site, size, and number of hemangiomas and the development of KMS [1]. Cutaneous and visceral hemangiomas have both been implicated in KMS. Work-up in the index patient revealed probable low-grade disseminated intravascular coagulation, but there was no evidence of microangiopathic hemolysis. Histologically, kaposiform hemangioendotheliomas and tufted angiomas are the most frequent lesions reported in KMS [1]. Treatment options include compression therapy for hemangiomas, surgical excision of large solitary vascular lesions (whenever feasible), or ligation/embolization of feeder vessels when lesions are inaccessible for surgery. Systemic steroids and interferon alfa have shown benefit when vascular lesions are extensive and not amenable to surgery or embolization.
